# Reduced graphene oxide surface modification with nanoparticles and its efficiency in removing crystal violet and malachite green dyes from wastewater

**DOI:** 10.1039/d5ra08749j

**Published:** 2025-12-08

**Authors:** Sumood Al-Hadeethy, Aws Z. Abdulmajeed, Ali M. Alalusi, Alauddin M. Mahdi, Wassan N. Hussain, Ibraheem A. Alrazaq

**Affiliations:** a College of Basic Education, Department of General Sciences, University of Anbar Iraq awsa.zabin@uoanbar.edu.iq; b College of Pharmacy, Tikrit University Iraq; c College of Education for Pure Sciences, Department of Chemistry, Tikrit University Iraq; d Salah Aldine Education Directorate Tikrit Iraq

## Abstract

Industrial dyes are among the most complicated contaminants, posing an increasing hazard to aquatic life if left untreated. Carbon nanoparticles are extremely effective in the adsorption process. As a result, the purpose of this study was to manufacture reduced graphene oxide from sunflower husks, an affordable raw material, and modify its surface with zinc oxide and copper oxide nanoparticles that were produced in an environmentally friendly method. This improved material was employed as a sustainable adsorbent to remove crystal violet and malachite green dyes from wastewater. The results revealed that the optimal medium for adsorption was basic, with an equilibrium time of 25 minutes at 35 °C. Kinetic experiments found that the adsorption follows pseudo-second-order kinetics and is consistent with the Langmuir-Isotherm model. The thermodynamic study showed that the process is spontaneous and endothermic, with increased system randomness. Crystal violet had a removal effectiveness of 95.6%, and malachite green had one of 96% from wastewater.

## Abstract 2

The research aimed to create a sustainable, efficient material for eliminating wastewater dyes. The results showed rapid and excellent efficiency, which may be used for water purification applications.

## Graphical abstract

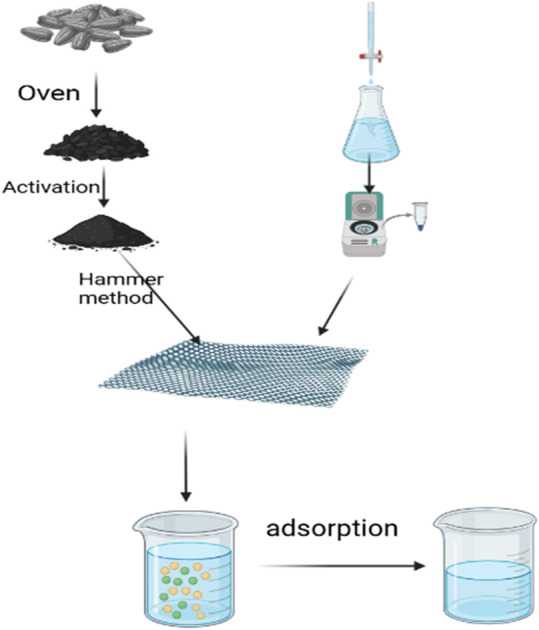

## 1. Introduction

Water resources worldwide face increasing threats from pollution caused by industrial and medical processes. Hospital wastewater is one of the most dangerous environmental pollutants because it contains complex chemical compounds such as pharmaceuticals, disinfectants, and stable organic dyes in aquatic systems. Among these pollutants, synthetic dyes are especially problematic due to their impact on water quality, reducing light penetration, and hindering photosynthesis. Additionally, some of these dyes are toxic or even carcinogenic to both organisms and humans.^1^ Dyes such as crystal violet and malachite green are widely used in biological laboratories as cell stains and diagnostic reagents. The dyes are released into wastewater streams in their residue state without proper treatment, leading to their concentration in the aquatic environment.^2^ Additionally, these dyes are to blame for mental illness, respiratory conditions, skin irritation, and vomiting.^3^

Crystal violet (CV), a triphenylmethane dye, is still very widely used in biological staining, dermatological uses, and commercial textile procedures, despite its mutagenic and carcinogenic traits. Taking in high amounts of the dye on the skin can lead to skin and digestive system irritation. Exposure to the dye may cause minor eye irritation and stinging when exposed to light.^4^ Malachite green is an organic compound that is a triphenylmethane class of dye and possesses enormous uses in industry and in biology. The chemical formula for malachite green is C_23_H_25_ClN_2_, and it can also be used as malachite green chloride. Malachite green is used mainly in textile dyeing and paper dyeing and as an antifungal and disinfectant in aquaculture for its use on fish for removing parasitic and fungal infections.^5^ Although beneficial in veterinary applications, malachite green is a harmful chemical and a potential carcinogen. Although identified in research to have significant toxic effects on aquatic organisms, it causes physiological and anatomical interruptions in fish, such as liver, gill, and reproductive tissue damage.^6^ It also possesses the capability of bioaccumulation and has the power to induce genetic alterations in microorganisms, posing risks to the equilibrium of aquatic ecosystems.^7^ Malachite green, as an environmentally highly toxic compound, has led most nations to prohibit or limit its application in aquaculture, particularly after residues of the compound were found in fish meat meant for human consumption. This dye is also used extensively in hospital laboratories for histological staining; therefore, it becomes a regular contaminant of wastewater in hospitals, which is discharged into aquatic environments by the effluent discharge from clinical and biochemical departments.^8–10^ When discharged to the environment, malachite green poses harmful effects on freshwater life forms such as fish, algae, and invertebrates. It is tightly fixed to cell proteins and enzymes, inhibiting significant processes such as cellular respiration and ATP synthesis.^11,12^ Various advanced technologies have been developed to remove these toxic dyes from wastewater, including adsorption, photocatalysis, membrane filtration, coagulation and flocculation, and advanced oxidation processes.^13^ Among water treatment technologies, adsorption stands as one of the most interesting methods of removing organic pollutants because it is easy, effective, and possesses lower operating costs in comparison with common methods such as chemical precipitation or advanced oxidation.^14^ Carbon nanomaterials have attracted the attention of researchers, mainly graphene oxide with a wide surface area and dense active functional groups, making it very effective in the adsorption of a wide range of organic pollutants.^15^ As the world has been moving towards more green processes, using agricultural waste to create nanomaterials has become more of a sustainable process. The sunflower husks that are a carbonaceous agricultural waste can be used as an inexpensive raw material for the sustainable production of graphene oxide, lowering solid pollution and upholding the concept of a circular economy.^16^ Within this perspective, this research aims to develop graphene oxide from sunflower seed shells and examine its ability to adsorb poisonous organic dyes in hospital wastewater, while exploring the influence of various operating parameters such as contact time, pH, and concentration of the dye, as well as temperature, to explore the mechanism of interaction between the adsorbent and the pollutant.

## 2. Experimental

### 2.1. Preparation of reduced graphene oxide

All chemicals were provided with a purity of >98% from Sigma-Aldrich and BDH. A procedure^17,18^ with some modifications was used to synthesize reduced graphene oxide from activated carbon. Twenty grams of sunflower husks were collected, washed thoroughly with distilled water, left to dry, then burned using a ceramic dish in a MemmertLab oven at 650 degrees Celsius for two hours. The charcoal was cooled to room temperature and ground thoroughly. Then, 1M HCl (Sigma-Aldrich) solution was added and mixed thoroughly and then placed in an oven at 750° for 3 hours. Five grams of dry charcoal, thoroughly washed with deionized water, were placed in a one-liter beaker, to which 3 grams of sodium nitrate were added. The beaker was then placed in an ice bath to maintain a constant temperature (0–5 °C). Then, 70 ml of concentrated sulphuric acid (98%) was added, and the mixture was stirred for 7 hours. Nine grams of potassium permanganate were gradually added to the mixture, ensuring that the temperature did not exceed 30 °C, and the mixture was left to stir for 24 hours. Then, 400 ml of deionized water was gradually added, and to stop the reaction, 10 ml of 30% hydrogen peroxide was added. The mixture was transferred to an ultrasonic device for 20 minutes, then the precipitate was separated by centrifugation and washed thoroughly with deionized water and dried thoroughly in an oven at 60 °C for 12 hours.

### 2.2. Zinc oxide nanoparticle synthesis

In a 500 mL beaker, 2 g of zinc acetate was mixed with 20 mL of deionized water and stirred constantly at room temperature. Using a pipette, 0.1 M ammonium hydroxide solution was gradually added until the pH reached 10. The solution was stirred for an hour before being placed in a drying oven at 60 °C for 12 hours. The precipitate was produced by centrifugation at 3800 rpm for 20 minutes. The precipitate was extensively cleaned to eliminate byproducts before being dried at 120 °C for one hour.^19^

### 2.3. Copper oxide nanoparticle synthesis

Copper oxide was prepared in an environmentally friendly method by placing 100 ml of copper sulfate (0.1 M) in a 500 ml container and leaving it on an electric stirrer. Then, a pipette was used to gradually add 5 milliliters of ascorbic acid as a reducing agent. Then, 0.1 M sodium hydroxide was added until the pH reached 12. The mixture was heated to 80 °C for half an hour, then the precipitate was collected by centrifugation, washed thoroughly with deionized water, and dried thoroughly using a drying oven at 100 °C for two hours.^20^

### 2.4. Modification of reduced graphene oxide surface by zinc oxide and copper oxide nanoparticles

The surface of rGO was modified using the Cheng method,^21^ with certain modifications. 1.5 g of rGO was dissolved in 100 ml of deionized water, along with 0.5 g of zinc oxide and 0.25 g of copper oxide, and then placed in an ultrasonic device for 20 minutes. The mixture was then allowed to stir for 24 hours at room temperature. The precipitate was produced by centrifugation and dried at 60 °C for 12 hours.

### 2.5. Characterization of synthesized nanoparticles

Various diagnostic techniques, such as FTIR (Shimadzu) and XRD (Gonio, 1.54 Å, 40 kV), were used to confirm the success of nanoparticle preparation and surface modification. The size of the particles was determined using the Debye–Scherer equation, and the distance between planes (*d*-spacing) was found using the Bragg equation.^22^ Morphological characterization was performed using a field emission scanning electron microscope (FESEM) and atomic force microscopy (SPMAA300, USA) to study the surface of the distinctive material. Atomic Force Microscopy analysis, a high-resolution technique, was utilized to investigate the surface topography, roughness, and other physical features of the adsorbent.

### 2.6. Adsorption process

#### 2.6.1. Determining the optimal conditions for the adsorption process

Adsorption experiments were set up with crystal violet dye and malachite green. Different dye concentrations were prepared to make a calibration curve using deionized water as the solvent. A Klab alpha spectrophotometer (USA) was used to determine absorbance. The optimal experimental parameters for the adsorption process were determined, including the optimum adsorbent weight, pH, temperature, and the effect of concentration and time on adsorption capacity. A shaking water bath was used to study these conditions, and the solution was then filtered using Whatman cellulose filter paper. Each experiment was repeated three times under identical conditions to ensure reproducibility and confirm the accuracy of the results. The adsorption capacity was calculated using eqn (1):1
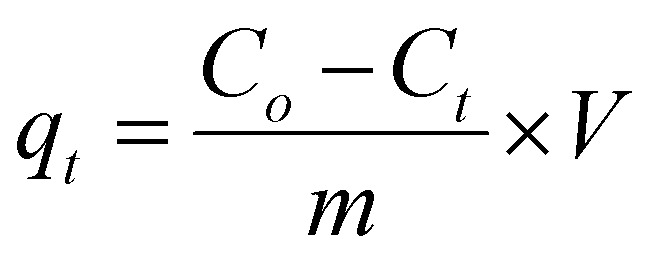
where *q* represents the adsorption capacity, *C*_o_ represents the initial concentration of the dye, *C*_*t*_ represents the concentration at equilibrium, *V* represents the volume of the solution, and *m* represents the mass of the adsorbent.

To calculate the percentage of adsorption, eqn (2) was used.2
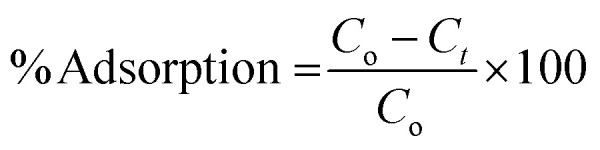


#### 2.6.2. Adsorption kinetics

The adsorption kinetics were studied over different time periods (5–35 minutes) in order to obtain equilibrium data. The optimum weight of the adsorbent was used, as well as the natural pH of the dye solution (20 ml) with a concentration of 4 mg L^−1^ for crystal violet dye and a concentration of 35 mg L^−1^ for malachite green dye, at a temperature of 30 °C. The first-order kinetic model eqn 3 and the second-order kinetic model eqn 4 were used.

3 ln (*q*_e_ − *q*_t_) = ln *q*_e_ − *k*_1_*t*

4
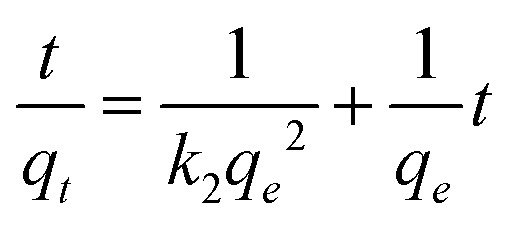
where *q*_e_ and *q*_*t*_ represent the adsorption capacity of the dyes at equilibrium and at different times, respectively, *k*_1_ and *k*_2_ are the adsorption rate constants for the first- and second-order pseudo reactions, respectively, and *t* is the reaction time in minutes.

#### 2.6.3. Adsorption isotherm

The isotherms of CV and MG dyes used in this study were studied using different concentrations of the adsorbate and the natural pH of the dye solutions and a solution volume of 20 ml at a temperature of 30 °C. Different adsorption models were used to describe the adsorption isotherm.^23,24^eqn (5) and (6) refer to Langmuir's isotherm model for the adsorption of a single layer on a surface, which occurs in areas that are energetically equivalent and homogeneously distributed, while eqn (7) refers to Freundlich's isotherm model for the adsorption of heterogeneous surfaces (energy-non-equivalent) and for a multi-molecular layer. Eqn (8) represents the Dubinin–Radushkevich isotherm model for heterogeneous surfaces and determines whether the adsorption is physical or chemical. Finally, eqn (10) refers to the Temkin isotherm model:5
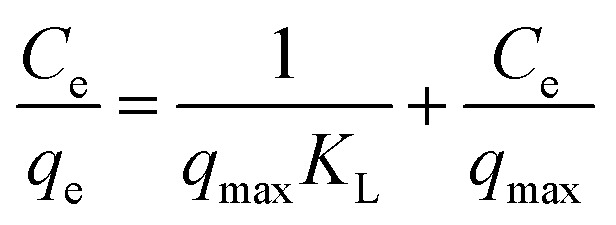
6
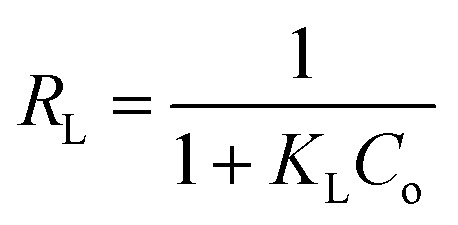
7
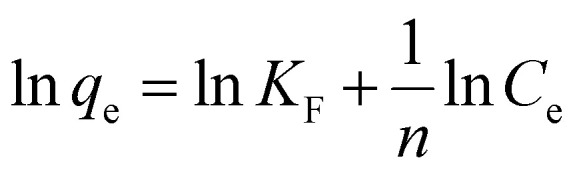

8 ln *q*_e_ = *q*_s_ − *Kε*^2^

9
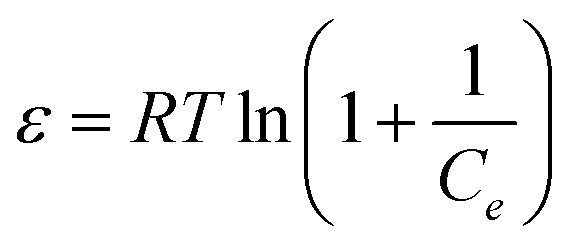
10
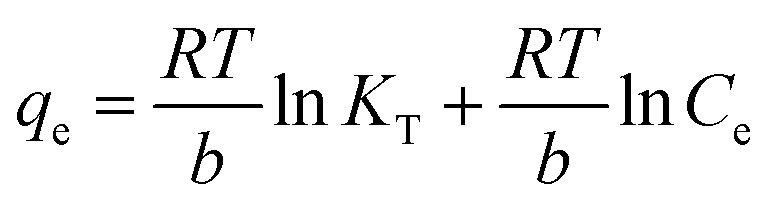
11
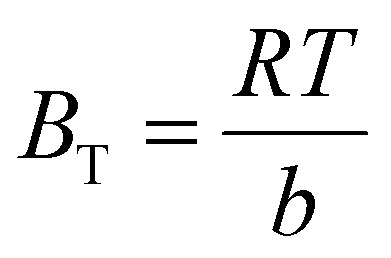
12
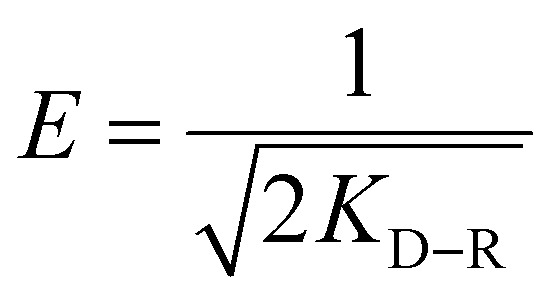
where *C*_e_ is the concentration at equilibrium, *q*_e_ is the adsorption capacity at equilibrium, *q*_max_ is the maximum adsorption capacity, *R* is the ideal gas constant, *T* is the temperature, *K*_L_ is the Langmuir constant, *K*_F_ and *n* are Freundlich constants, *q*_s_ is the maximum adsorption capacity, *K* is the Dubinin–Radushkevich constant, *ε* Bolani constant which is calculated from eqn (9), *B*_T_ and *K*_T_ are the Temkin equilibrium constants, and *E* represents the average free energy of adsorption (kJ mol^−1^) per adsorbate molecule at the moment of transition from the liquid phase to the solid phase.

#### 2.6.4. Determination of thermodynamic constants

In order to calculate the thermodynamic properties of the dye adsorption process in the current study, the Van't Hoff equation was used to calculate the standard adsorption enthalpy eqn 13, while eqn (15) and (16) were used to calculate the standard free energy and entropy of the standard adsorption process:13
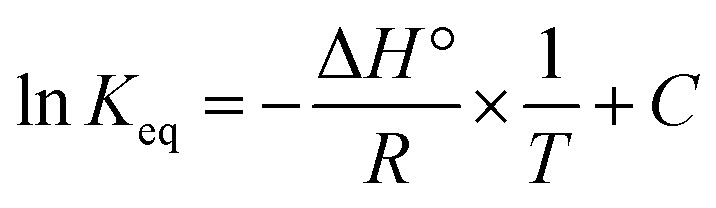
14
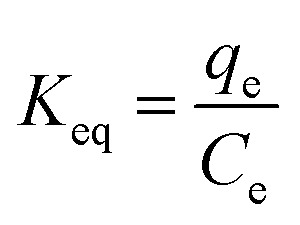

15 Δ*G*° = − *RT* ln *K*_eq_

16 Δ*G*° = Δ*H*° − *T*Δ*S*°

where *C* represents the Van't Hoff constant, *K*_eq_ represents the equilibrium constant, *H*° represents the standard enthalpy, Δ*S*° represents the standard entropy, and Δ*G*° represents the standard free energy.

### 2.7. Removing dyes from wastewater

After determining the optimal conditions for the adsorption process *in vitro*, adsorption was performed to remove the dyes used in the current study from the wastewater of Hadeetha General Hospital in Anbar Province, Iraq. The adsorption capacity and percentage of adsorption were calculated as in eqn (1) and (2) using 0.05 g of the prepared adsorbent after 25 min of incubation at 35 °C.

## 3. Results and discussion

### 3.1. Characterization of synthesized nanoparticles

**Fig. 1:**
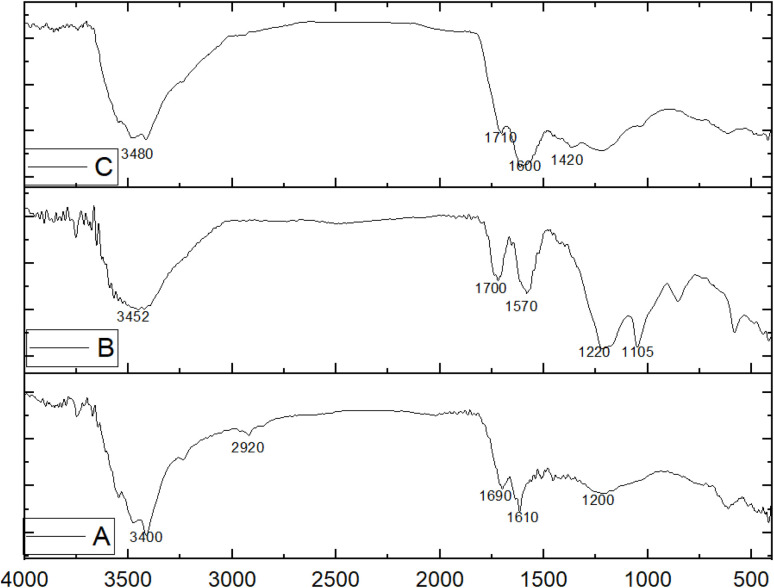
Infrared spectrum (A) Activated carbon, (B) Reduced graphene oxide, (C) rGO-ZnO-CuO.

In order to better understand the structure, morphology, and composition information of the prepared particles, they are characterized using FTIR, XRD, SEM, and AFM, respectively. FTIR provides information on the identity of functional groups, and the results of infrared spectrum analysis showed distinctive peaks in activated coal, indicating a number of active groups, as shown in Fig. 1-A, where the sharp peak at the wave number 3400–3500 cm^−1^ indicates the hydroxyl alcohol group, while the peak at 2920–2850 cm^−1^ indicates the C–H group of alkane, the peak at 1050–1100 cm^−1^ indicates the C–O alcohol group, and the peak at 1620 cm^−1^ indicates the C=C group. While the peak at 1699 cm^−1^ corresponds to the carbonyl group. These results indicate that activated coal is an aromatic with limited functional groups. Figure. (1-B) shows the results of the infrared spectrum analysis of reduced graphene oxide prepared from activated coal. The spectrum shows the presence of a number of distinctive peaks and the disappearance of others. The broad peak at 3452 cm^−1^ indicates the hydroxyl group present on the surface of reduced graphene oxide, and the peak at 1718 cm^−1^ is attributed to the carbonyl group. The peak at 1570–1620 cm^−1^ corresponds to the C=C bond of the cyclic alkyne, and the peak at 1105–1100 cm^−1^ corresponds to the C–O bond, while the peak at 1220–1260 cm^−1^ corresponds to the C–O–C bond of the epoxide. This confirms the presence of alcoholic and epoxy groups that belong to reduced graphene oxide compared to activated coal, which is similar to previous studies.^25,26^Figure. (1-C) shows the results of infrared spectrum analysis of reduced graphene oxide after modifying its surface with zinc oxide and copper oxide, where the peak width decreased at a wavelength of 3480 cm^−1^, which is due to the shift of the hydroxyl alcohol group. The peak at a wavelength of 1700 cm^−1^ is attributed to the carbonyl group, and the peak at a wavelength of 1600 cm^−1^ is attributed to the C=C group of the cyclic alkyne. The spectrum also shows an increase in the metal–oxygen bond ranges due to the presence of zinc oxide and copper oxide within the range of 620–420 cm^−1^, where the peak at 1420 cm^−1^ indicates Zn–O and the peak at 1615 cm^−1^ indicates CuO, which indicates modification of the surface of reduced graphene oxide by zinc oxide and copper oxide. The peak at 3400–3500 cm^−1^ is moderate in coal and strong in rGO and decreases after modification of the rGO surface, which indicates a coordinate bond between hydroxyl groups and copper or zinc, reducing their degrees of freedom and thus reducing vibration. Meanwhile, the carbonyl group at 1720–1740 cm^−1^ is weak in activated coal but distinct in rGO and decreases after surface modification due to the effect of coordination with zinc and copper as well, while the C–O–C and C–O groups are unclear in activated coal but distinctive in rGO and decrease after modification due to coordination with the oxides used in surface modification. In addition, the M – O bands increase after surface modification, which confirms the introduction of Zn–O and CuO groups.

**Fig. 2:**
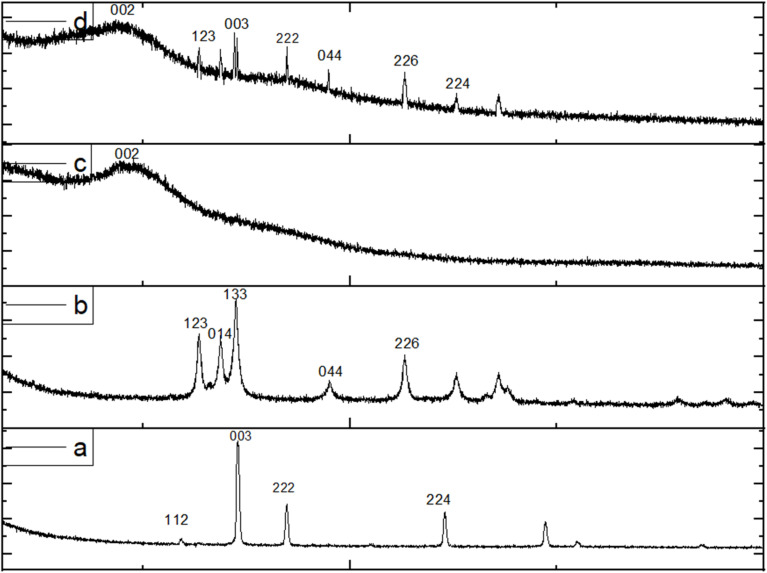
X-ray diffraction (a) copper oxide, (b) zinc oxide, (c) reduced graphene oxide, (d) rGO-ZnO-CuO.

The results of X-ray diffraction analysis of copper oxide nanoparticles (Fig. 2a) showed the appearance of main peaks at 2*θ* (36.5, 42.4, 52.4, and 61.3°), indicating the successful synthesis of Cu_2_O nanoparticles according to JCPDS card number 96-900-7498.^27^ The XRD measurement also shows no interference between other peaks, confirming the high purity of copper oxide. The lattice constant (a) value reached 7.40 Å, the microstrain (*ε*) 6.9 × 10^−4^, and the crystal size ranged between 1.6 and 7.5 nm. The XRD pattern of zinc oxide (Fig. 2b) showed the appearance of main peaks at 2*θ* (31.7, 34.3, 36.2, 47.4, 56.5, 62.7, 67.8, 69, and 77°). These results are consistent with JCPDS card number 96-900-8878.^28^ The high purity of the compound prevents the appearance of additional peaks. The lattice constant (a) reached 10.75 Å, the microstrain was 0.0514, and the crystal size ranged between 20.5 and 50.6 nm. In addition, the results shown in Figure 2c revealed a broad peak at 2*θ* of 26–24°, which is attributed to reduced graphene oxide, consistent with previous studies.^29–31^ The distance between the sheets reached 0.34 nanometers. Furthermore, the results of XRD analysis of the rGO-ZnO-CuO nanocomposite (Fig. 2d) showed a distinctive broad peak at 2*θ* 26–24° corresponding to rGO, in addition to several sharp, high-density peaks corresponding to copper oxide and zinc oxide, which is consistent with previous studies.^32^ This confirms the success of modifying the surface of rGO with zinc oxide and copper oxide particles.

**Fig. 3:**
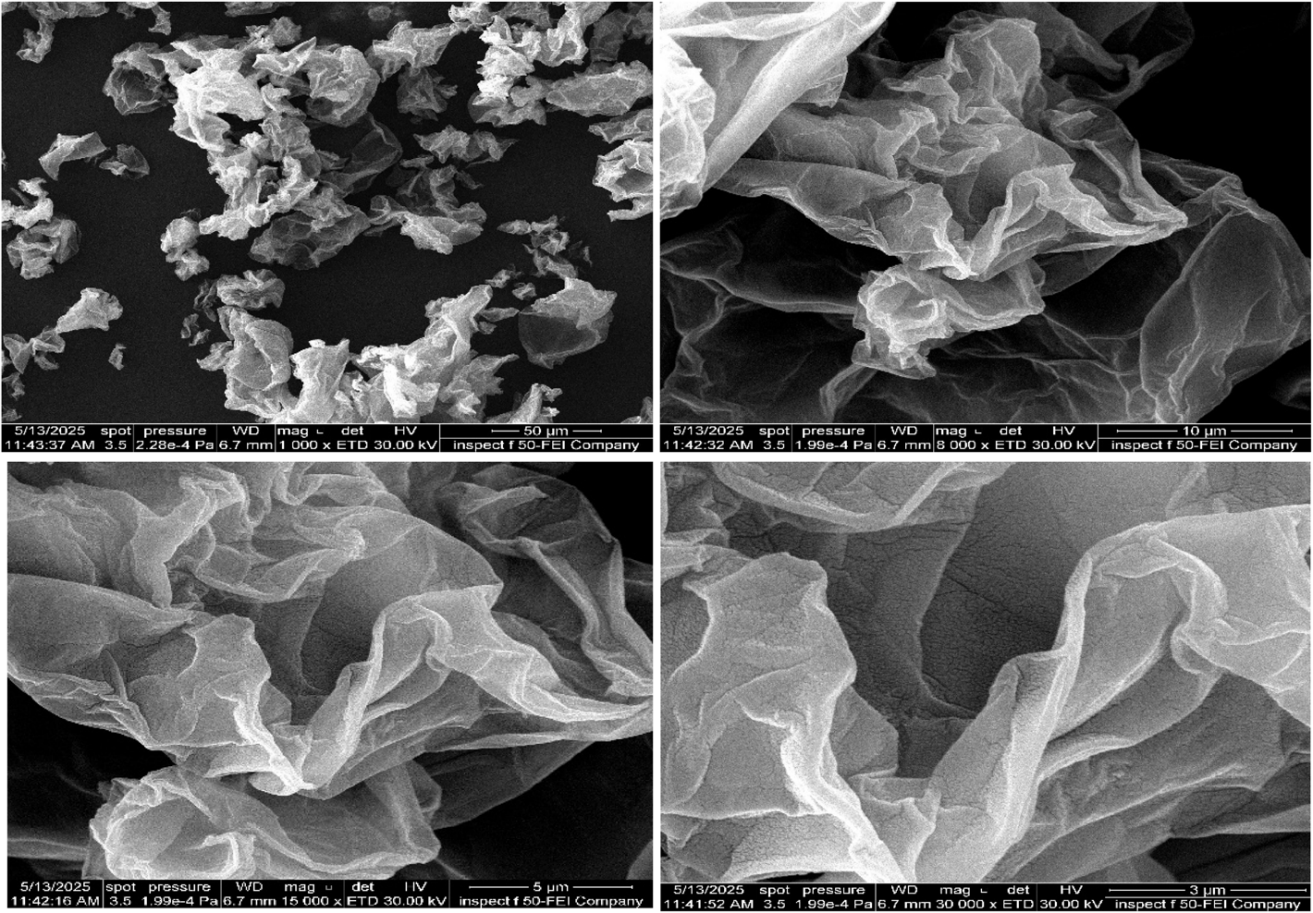
FESEM analysis of rGO.

FESEM technology is used to determine the size and shape of reduced graphene oxide and rGO-CuO-ZnO nanocomposite, as shown in Figure (3), which shows curled and folded sheets of reduced carbon oxide, which closely resemble thin folded sheets, a characteristic feature of rGO due to its inherently two-dimensional nature. Larger clusters of curled sheets can be seen at a magnification of 2000×, which proves that the material is not flat. These morphological and structural characteristics are important for understanding the behavior of rGO in the adsorption process.

**Fig. 4:**
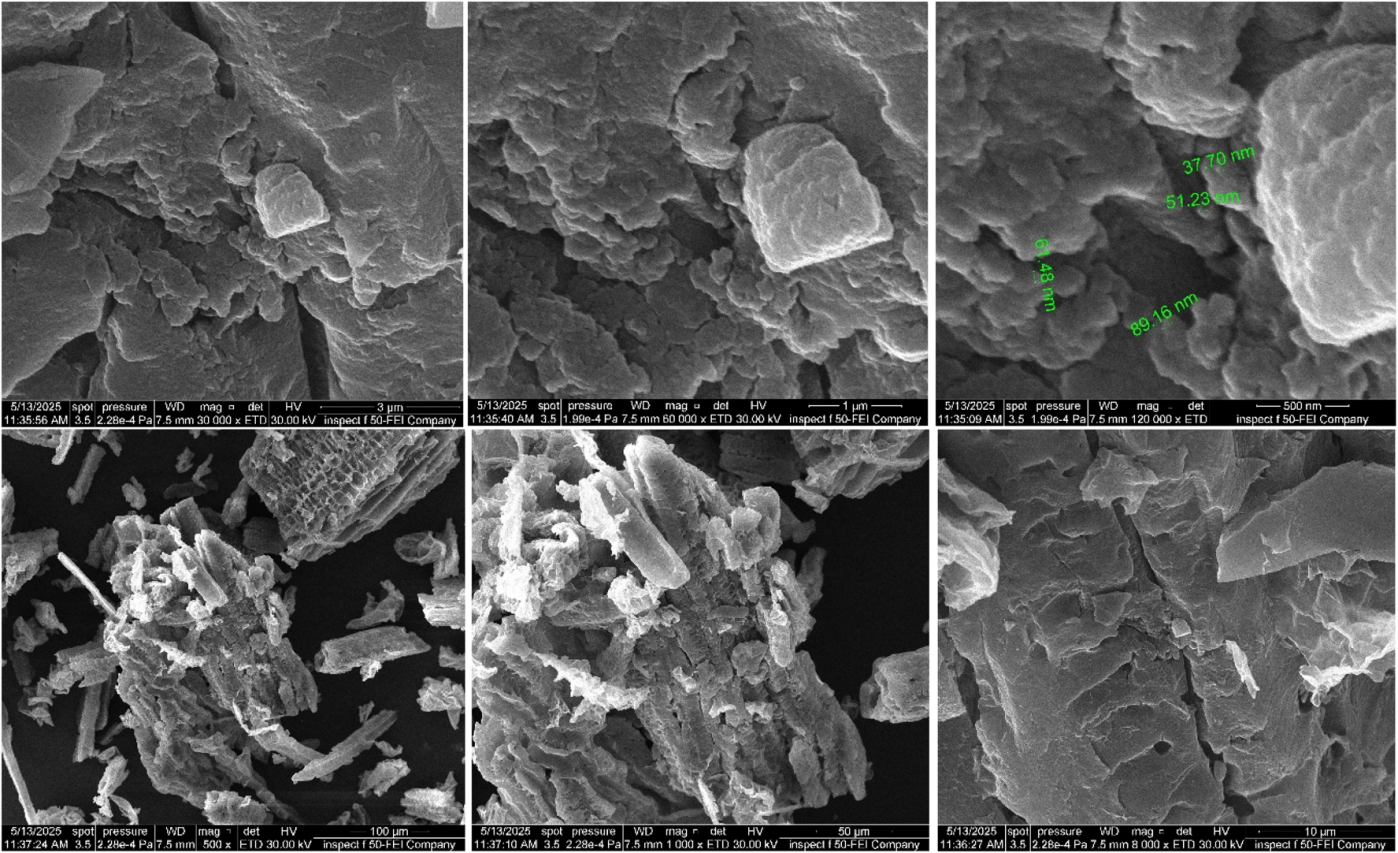
FESEM analysis of rGO-CuO-ZnO nanocomposite.

While the surface appears rough in Fig. 4, layered aggregates of reduced graphene oxide can be seen with nanostructures distributed on top of them, confirming the modification of the rGO surface by zinc oxide and copper oxide particles. Where deposited and relatively distributed nanoparticles can be seen on the surface of reduced graphene oxide, as the nanoparticles can be observed carried on the edges of the sheets and in the interstitial spaces. These dimensions of 37.70 nm, 51.23 nm, and 89.16 nm indicate the size of the zinc oxide and copper oxide nanoparticles that were successfully deposited on the surface of the reduced graphene oxide. In other words, the resulting material has a highly porous and tortuous surface, which may increase its adsorption properties.

**Fig. 5:**
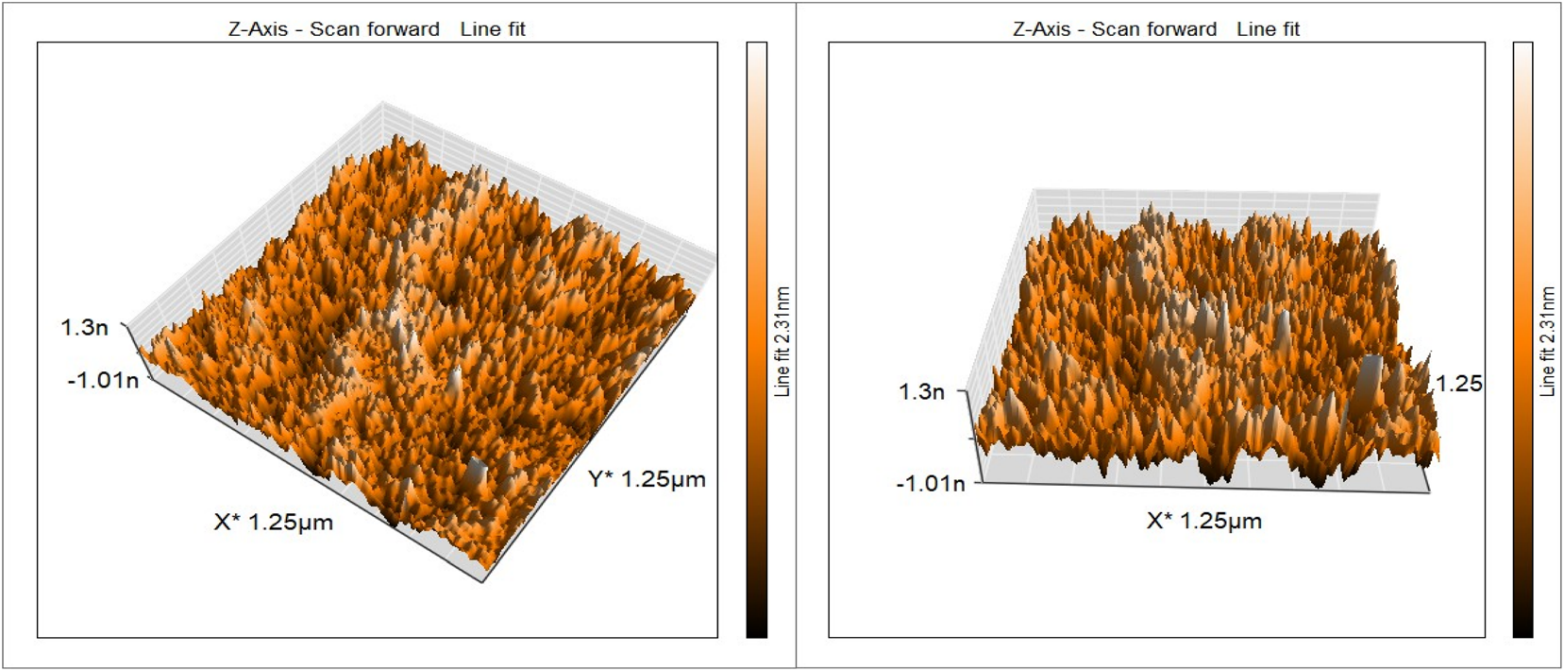
AFM analysis of rGO-CuO-ZnO nanocomposite.

AFM images are used to determine topographical characteristics and evaluate surface roughness. AFM analysis confirms that the material has a very rough surface topography, with a Root Mean Square Roughness (RMS) value of 2. This is evidence of the good distribution of nanoparticles on the surface of reduced graphene oxide, which prevents them from agglomerating into large clusters, consistent with the expected properties of reduced graphene oxide doped with zinc oxide and copper oxide particles. As shown in Figure (5), instead of obtaining a relatively flat surface, these particles are deposited on the surface of the reduced graphene oxide, creating bumps and ridges that confirm that the material is irregular in structure, which is consistent with the expected appearance of reduced graphene oxide doped with zinc oxide and copper oxide. Graphene oxide sheets also typically exhibit wrinkles and folds on the surface, which contribute to increased roughness.

### 3.2. Optimal conditions for dye adsorption in the aqueous phase

The properties obtained through the techniques used in diagnosing the adsorbent indicate that the nanocomposite is good for removing cationic dyes and pollutants. The calibration curve was drawn using deionized water as the blank solution and concentrations ranging from 0.4 to 5.0 mg L^−1^ for crystal violet dye (*λ* = 588 nm) and malachite green dye (*λ* = 618 nm). The results showed a straight linear equation for crystal violet dye, *y* = 0.2807*x* + 0.0567, with a value of *R*^2^ = 0.9938. While for the malachite green dye, the straight-linear equation was *y* = 0.1581*x* + 0.0624, with an *R*^2^ value of 0.9936, as shown in (S1).

**Fig. 6:**
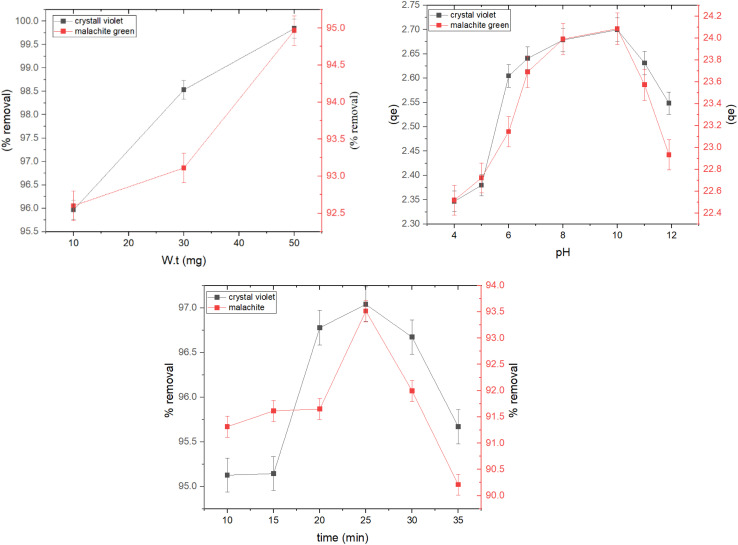
Effect of rGO-ZnO-CuO nanocomposite dosage, contact time, and pH on the adsorption process.

Studying the dose of the adsorbent is necessary to avoid wasting the material once equilibrium is reached. The effect of the adsorbent doses on adsorption efficiency was studied, and the results showed that the percentage of adsorption increases with increasing doses of the prepared nanocomposite (0.01–0.05 gm), using an initial concentration of 4 mg L^−1^ for crystal violet dye and 36 mg for malachite green dye, at a temperature of 30 °C and an equilibrium time of 25 minutes, as shown in Fig. 6, where the adsorption efficiency increases linearly with the increase in the amount of adsorbent due to the increase in the number of active sites available for adsorption and the increase in the surface area for interaction between the dyes and the adsorbent with the increase in the adsorbent dose, which is consistent with previous studies,^33,34^ these results confirmed that the optimal dose was 0.05 gm for the adsorption of dyes from aqueous solutions.

The pH of the solution is one of the main parameters controlling the adsorption of dyes from wastewater onto the adsorbent nanocomposite. The pH value of wastewater is an important factor for the hydroxyl, amine, and carboxyl functional groups on the surface of rGO, as it creates an attractive force between the adsorbent and the cationic dyes. The effect of pH on the adsorption of crystal violet dye and malachite green dye at different pH values (4–12) was studied. The results showed that the best dye removal rate was in the basic medium at a pH of 10 using 30 mg of the adsorbent at a temperature of 30 °C and an equilibrium time of 25 minutes. The removal rate for crystal violet dye was 99.93%, while the removal rate for malachite green dye was 99.89%. Fig. 6 shows that the adsorption capacity increases continuously with increasing pH values until it reaches an optimal pH of 10. Due to the competition between cationic dye molecules and ionized hydrogen ions in the acidic medium to bind to the active sites of the prepared material, the surface becomes positively charged, which prevents dye molecules from approaching and binding to the surface due to electrostatic repulsion. This effect decreases with the decrease in hydrogen ion concentration when moving to a neutral and then basic medium, reducing competition and increasing electrostatic attraction between dye molecules and anionic active sites on the surface, thereby increasing the adsorption ratio. These results are consistent with previous studies that showed that the optimal pH for the adsorption of violet crystal dye and green malachite was 10.^34,35^

In addition, the effect of contact time was studied using an initial concentration of 4 mg L^−1^ of crystal violet dye and 36 mg of malachite green dye at a temperature of 30 °C and at different time intervals (5–35 minutes). The results showed that equilibrium was reached after 25 minutes with a percentage of removal of 97% for crystal violet dye and 93.5% for malachite green dye, while a previous study indicated a removal rate of 89.7% with a contact time of 30 minutes using 0.5 g of peanut shells for crystal violet dye.^36^ Another study indicated that the percentage of green malachite dye adsorption reached 98.2% at an equilibrium time of 60 minutes when using nano-bentonite saturated with magnesium oxide.^37^

It can be seen from Fig. 6 that the percentage of removal increases with increasing contact time due to the large number of active sites on the surface, while the percentage decreases after 25 minutes due to the saturation of the active sites with dye molecules that are weakly bound to the surface, which reduces the dye removal rate.^38^

### 3.3. Adsorption kinetics of violet crystal dyes and green malachite on the surface of rGO-ZnO-CuO nanocomposite

Studying adsorption kinetics is a suitable method for determining the rate of dye removal from an aqueous solution. The adsorption kinetics of crystal violet and malachite green dyes were studied by applying a pseudo-first-order model (Lagrangian model), which links the adsorption rate to the number of vacant sites available for adsorption on the surface, *i.e.*, adsorption depends on diffusion in the solution, and the binding between the adsorbate and the active sites depends on the formation of physical bonds such as van der Waals forces, and the pseudo-second-order model, which assumes that the adsorption process occurs through chemical bonds. The pseudo-second-order model showed a better *R*^2^ value compared to the pseudo-first-order model. The calculated and experimental adsorption capacity *q*_e_ values are consistent with the pseudo-second-order model, as shown in Table 1, which confirms the suitability of the pseudo-second-order model for the adsorption of dyes on the surface of rGO-ZnO-CuO nanocomposite, *i.e.*, the specific step of the reaction depends on the formation of ionic bonds and π–π bonds between the molecules of the adsorbate and the active sites on the adsorbate surface.^39^ These results are consistent with the findings of Abbas,^36^ who reports that the absorption of violet crystal dye follows pseudo-second-order kinetics. Meanwhile, Ullah^40^ indicated that the adsorption of malachite green dye follows pseudo-first-order kinetics using mesoporous natural inorganic clay.

**Table 1:** Kinetic parameters for CV and MG dyes removal

Model	Parameter	Dye	
		Crystal violet	Malachite green
Pseudo-first order	*q*_e_ (mg g^−1^)	1.896	0.618
	*R* ^2^	0.753	0.832
	*k*_1_ (min^−1^)	0.198	0.016
Pseudo-second order	*q*_e_ (mg gm^−1^)	2.678	22.371
	*R* ^2^	0.99	0.99
	*k*_2_ (gm mg^−1^ min^−1^)	2.678	0.644

The results also showed that the rate constant for the adsorption process *k*_2_ is 2.678 for crystal violet dye and 0.644 for malachite green dye. These results support the conclusion that the best description of the adsorption kinetics is pseudo-second-order diffusion mechanisms with contributions from surface interactions.^41^ These results indicate that the adsorption process occurs in three successive stages. In the first stage of adsorption, dye molecules migrate from the solution to the surface of the rGO-ZnO-CuO nanocomposite, forming a boundary layer on the surface. In the second stage of adsorption, the dye molecules slowly migrate to settle on the surface and pores in a specific order. The final stage occurs more slowly, depending on the shape and diameter of the pores, as the dye molecules fill the pores of the rGO-ZnO-CuO nanocomposite material.

### 3.4. Isotherms of adsorption of violet crystal and green malachite dyes on the surface of rGO-ZnO-CuO nanocomposite

**Fig. 7:**
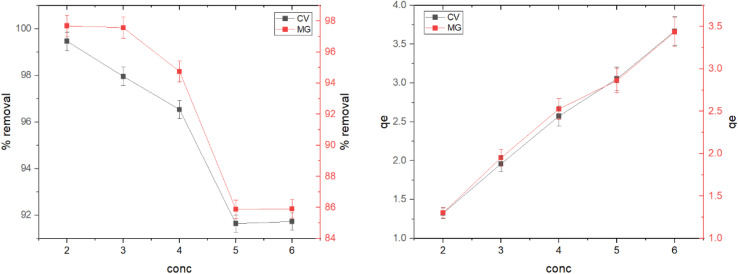
Effect of time on removal percentage and adsorption capacity for CV and MG dyes.

The initial concentration of dyes is a key factor in improving adsorption behavior. The effect of the initial concentration of dyes on the adsorption process was studied using different initial concentrations of dyes at a temperature of 30 °C, with an equilibrium time of 25 minutes and a dose of 30 mg of rGO-ZnO-CuO nanocomposite. Fig. 7 shows the effect of the initial concentration of dyes on the percentage and adsorption capacity. It can be seen that the percentage of adsorption decreases with an increase in the initial concentration of dyes, while the adsorption capacity increases linearly with the concentration of dyes. The percentage of adsorption reached 99% at a concentration of 2 mg L^−1^ for crystal violet dye and 97.7% for malachite green dye under the same conditions. This can be explained by the decrease in the number of active sites available on the surface as the concentration increases.^39^

Adsorption isotherms efficiently describe the interaction between the dyes and the active sites on the surface in equilibrium. Initial concentration data for crystal violet and malachite green dyes were used to describe the adsorption isotherm using the Langmuir, Freundlich, Dubinin–Radushkevich (D–R), and Temkin models. The Langmuir model assumes that adsorption occurs as a result of covering the surface with a single layer and uniform distribution of the dye on specific homogeneous sites on the surface, and that each dye molecule bound to the surface of the adsorbent has a constant adsorption energy. (S2 and S3) show the linear relationship for Langmuir. While Freundlich's model assumes that the dye is adsorbed on heterogeneous active surface sites through a multi-layer adsorption process with an exponential decrease in adsorption energy as the active sites are occupied. Freundlich's linear relationship is illustrated in S2 and S3. While the Timken model proposes that the adsorption energy per dye molecule decreases linearly with increasing surface coverage due to the indirect interactions of dye molecules and active sites. While the Dubinin–Radushkevich (D–R) relationship assumes that the adsorption process occurs inside the pores and not only on their outer surface, according to Polanyi's theory, and that the adsorption energy is distributed homogeneously on heterogeneous surfaces. Table 2 shows the results of the four models and their corresponding correlation coefficients (*R*^2^).

**Table 2:** Adsorption isotherm constants of crystal violet and malachite green dye by rGO-ZnO-CuO nanocomposite

Model		Dye	
		Crystal violet	Malachite green
Langmuir	*q*_max_ (mg g^−1^)	3.244	3.484
	*K*_L_ (L mg^−1^)	0.0028	0.0062
	*R*_L_ (L mg^−1^)	0.0084	0.021
	*R* ^2^	0.996	0.981
Freundlich	*n*_f_	3.98	3.669
	*K*_f_ (mg g^−1^)	4.095	3.5001
	*R* ^2^	0.98	0.891
Dubinin–Radushkevich	*q*_s_(mg g^−1^)	1.205	1.2167
	*E* (kJ mol^−1^)	8.45	7.071
	*R* ^2^	0.919	0.945
Temkin	*B*_T_(J mol^−1^)	1539.81	4220.72
	*K*_T_(L mg^−1^)	45.841	253.91
	*R* ^2^	0.817	0.927

Based on the *R*^2^ values, the Langmuir model is the best fit for the results obtained, with an *R*^2^ value of 0.996. These results confirm that the adsorption process occurs chemically in a single layer with uniform distribution at homogeneous sites and a constant adsorption energy, as each molecule is associated with only one active site, and the maximum capacity for covering the single layer is 3.24 mg g^−1^ for crystal violet dye and 3.48 mg g^−1^ for malachite green dye. However, the Timken and Dubinin–Radushkevich models cannot be applied to describe the adsorption process because the *R*^2^ value is far from one. The Langmuir constant indicates that the binding forces between the dyes and the active sites are strong, which is consistent with the results of the kinetic study, which proved that adsorption occurs through strong chemical bonds and not through weak physical bonds. The *R*_L_ constant calculated from the *K*_L_ value, which describes the difficulty of adsorption, indicates that the adsorption process occurs easily (0.0084 for crystal violet dye, 0.021 for malachite green dye), *i.e.*, it falls within the preferred range, confirming the suitability of the Langmuir model. The results of our current study are consistent with those of Sultana,^42^ who reported that the adsorption process of crystal violet obeys the Langmuir model when using coconut shell powder, and El Naeem^43^ when using sugar cane bagasse, while Abbas^36^ indicated that the adsorption process of crystal violet follows the Freundlich model when using a low-cost adsorbent from peanut shells. In addition, previous studies have indicated that the adsorption process of malachite green dye follows the Langmuir model when using different adsorbents.^40,44^

### 3.5. Thermodynamic study of the adsorption of violet crystal and green malachite dyes on the surface of rGO-ZnO-CuO nanocomposite

**Fig. 8:**
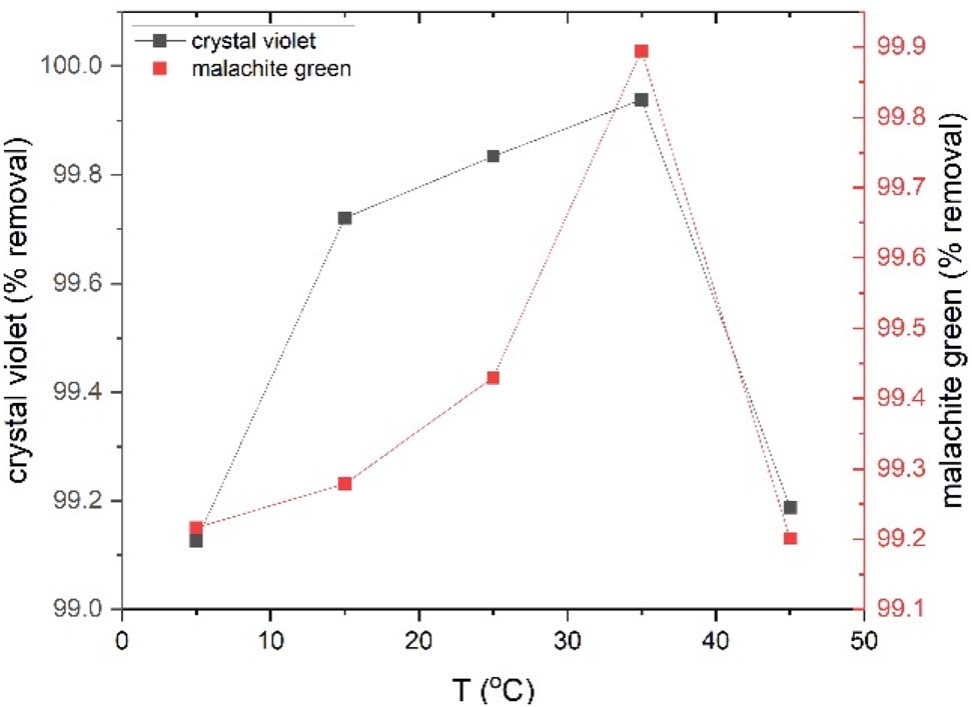
Effect of temperature on adsorption removal percentage for CV and MG dyes.

The effect of temperature on the adsorption capacity of CV dye and MG on rGO-ZnO-CuO nanocomposite at different temperatures was studied, as shown in Fig. 8. The results showed that the percentage of dye removal increases with increasing temperature due to the increase in the kinetic energy of the dye molecules in direct proportion to the increase in temperature, which causes an increase in collisions with the active sites on the surface, *i.e.*, the adsorption process is endothermic. The best adsorption ratio was achieved at a temperature of 35 °C, reaching 99.93% for crystal violet dye and 99.89% for malachite green dye, while a temperature higher than 35 °C leads to the migration of dye molecules from the active sites on the surface, thus reducing the percentage of adsorption.

To better understand the direction and type of forces affecting the adsorption process, the thermodynamic functions of adsorption were calculated. The Van't Hoff model (S4) was applied to calculate Δ*H*° based on temperature effect data, while Δ*G*° and Δ*S*° were calculated using eqn (15) and (16). Table 3 shows the results of the thermodynamic constants, where the positive value of enthalpy indicates that the adsorption process is endothermic. And its value is higher than 40 kJ mol^−1^, which confirms the kinetic and isothermal data of the study, which indicated that the adsorption process is chemical adsorption.^45^ While the positive entropy value indicates that the adsorption process is accompanied by an increase in the randomness of the system due to the increased collision rate of crystal violet and malachite green dye molecules with the active sites on the surface in the layer between the liquid and solid phases, in addition to the attraction of dye molecules by active sites and structural changes in dye molecules when they bind to the active site, as dye molecules have many possibilities to find a suitable adsorption site when the solvent molecules are displaced before adsorption. As dye molecules are present in large numbers as free ions near the active site compared to solvent molecules, causing increased randomness between the liquid and solid phases and thus increasing adsorption. Furthermore, the negative Δ*G*° values indicate that the adsorption process is thermodynamically spontaneous at different temperatures, and the decrease in free energy with increasing temperature indicates that adsorption improves with increasing temperature. These results are consistent with previous studies.^46–49^

**Table 3:** Thermodynamic parameters for CV and MG dyes removal

Temperature (°K)	Δ*H*°(kJ mol^−1^)		Δ*S*°(kJ mol^−1^ K^−1^)		Δ*G*°(kJ mol^−1^)	
	Crystal violet	Malachite green	Crystal violet	Malachite green	Crystal violet	Malachite green
278	60.719	43.793	0.255	0.195	−9.998	−10.252
288					−13.102	−10.821
298					−14.851	−11.782
308					−17.909	−16.505

### 3.6. Removing dyes from wastewater

Wastewater is highly complex, and since the goal was to remove crystal violet and malachite green dyes from wastewater most easily and efficiently, as the particle size decreases, the surface area increases, and as the surface area increases, the amount of absorbed material also increases. As shown by the XRD results, the rGO-ZnO-CuO nanocomposite has a nanoscale size and therefore a large surface area. In addition, there are functional groups after modification, which increases the interaction between the adsorbent and the adsorbate.

After determining the appropriate laboratory conditions for the adsorption of crystal violet and malachite green dyes, these conditions were applied to remove the dyes from the wastewater of Hadeetha General Hospital in Anbar Province, Iraq. The results showed that the removal rate using 30 mg of the prepared nanocomposite reached 95.6% for crystal violet dye and 96% for malachite green dye, with an adsorption capacity of 1.73 mg g^−1^ for CV dye and 4.18 mg g^−1^ for MG dye. Whereas the maximum removal rate for crystal violet dye using alfalfa stem powder in a previous study was 92%, with an adsorption capacity of 1.95 mg g^−1^.^50^ In another study, lemon peel was used as an adsorbent for crystal violet dye,^51^ with a removal rate of 89%, while the removal rate of malachite green dye using zinc tungstate (ZnWO_4_) nanoparticles was 74%.^52^ The reason for the reduction in the removal rate of dyes in wastewater compared to laboratory conditions is due to the high complexity of wastewater, where competition occurs between metal ions and organic compounds for binding to the active site on the surface.

## 4. Conclusion

During the current study, reduced graphene oxide was prepared in a cost-effective manner, and its surface was modified with zinc oxide and copper oxide nanoparticles. The prepared nanocomposite was used as an adsorbent to remove crystal violet and malachite green dyes from wastewater. The nanocomposite showed high efficiency in removing dyes, with a removal rate of 99.9% in a basic medium and an optimal equilibrium time of 25 minutes at a temperature of 35 °C. The kinetic study also proved that the preferred adsorption process is of the chemical type that follows pseudo-second-order kinetics, which is confirmed by the isothermal results that showed that the Langmuir model shows a high correlation with the current results. The thermodynamic results also indicated that the adsorption process is spontaneous at different temperatures, endothermic, and accompanied by an increase in the randomness of the system. The percentage of dye removal from wastewater reached 95.6% for crystal violet dye and 96% for malachite green dye, confirming the promising future of the nanocomposite prepared in the adsorption process compared to other specialized materials.

## Author contributions

All authors contributed to the preparation, conceptualization, design, data collection, and analysis of the study's material and approved the final version of the publication.

## Conflicts of interest

The authors affirm that they have no relevant financial interests or personal relationships that could have influenced the work presented in this paper.

## Supplementary Material

RA-015-D5RA08749J-s001

## Data Availability

Data will be available when request. Supplementary information (SI) is available. See DOI: https://doi.org/10.1039/d5ra08749j.
