# Supplementary material for: Reduced graphene oxide surface modification with nanoparticles and its efficiency in removing crystal violet and malachite green dyes from wastewater
Source: RSC Adv. 2025 Dec 8;15(56):48509–20. doi: 10.1039/d5ra08749j (PMC12683654; doi:10.1039/d5ra08749j)
Supplement: RA-015-D5RA08749J-s001 [file RA-015-D5RA08749J-s001.pdf]

Supplementary Information (SI) for RSC Advances.
This journal is © The Royal Society of Chemistry 2025

Supplementary data:

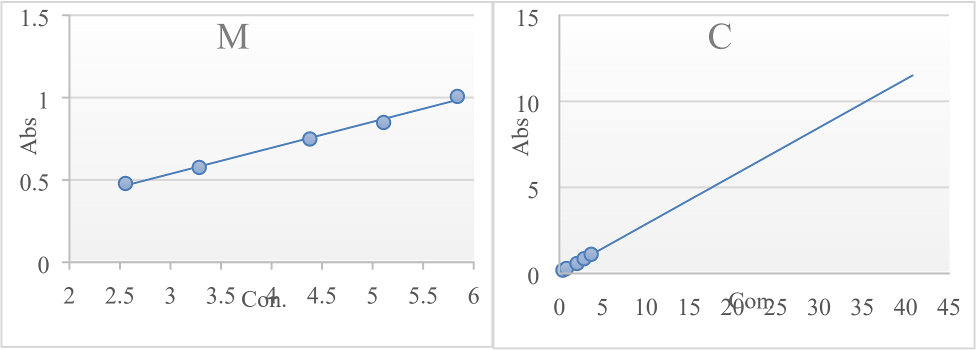

S1 standard curves of CV and MG dyes

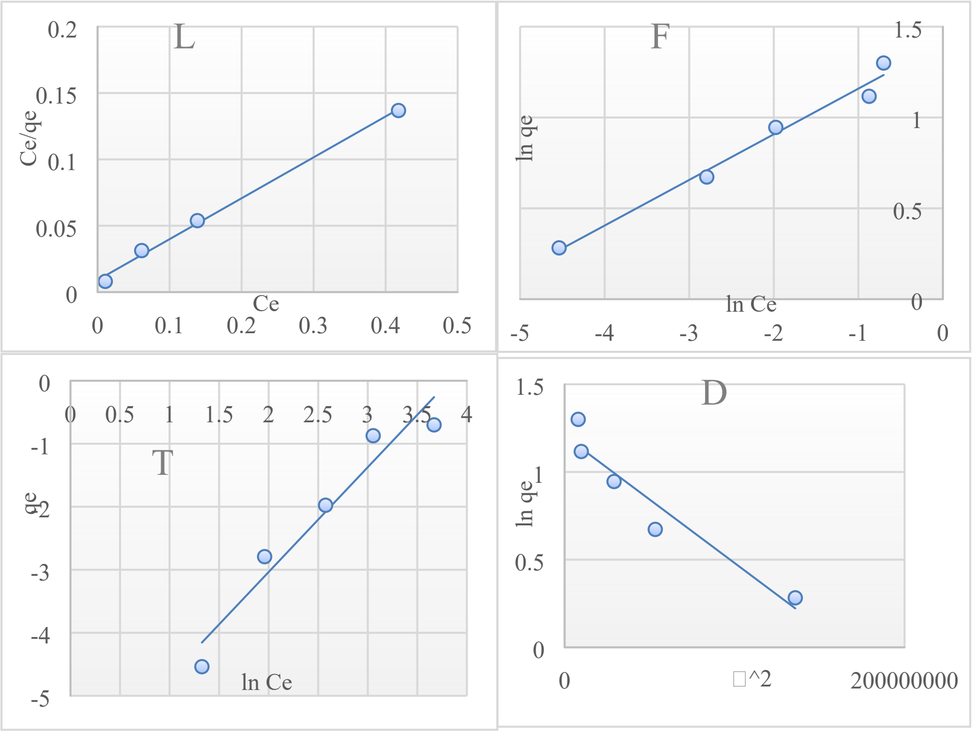

S2 Adsorption isotherm for CV dye

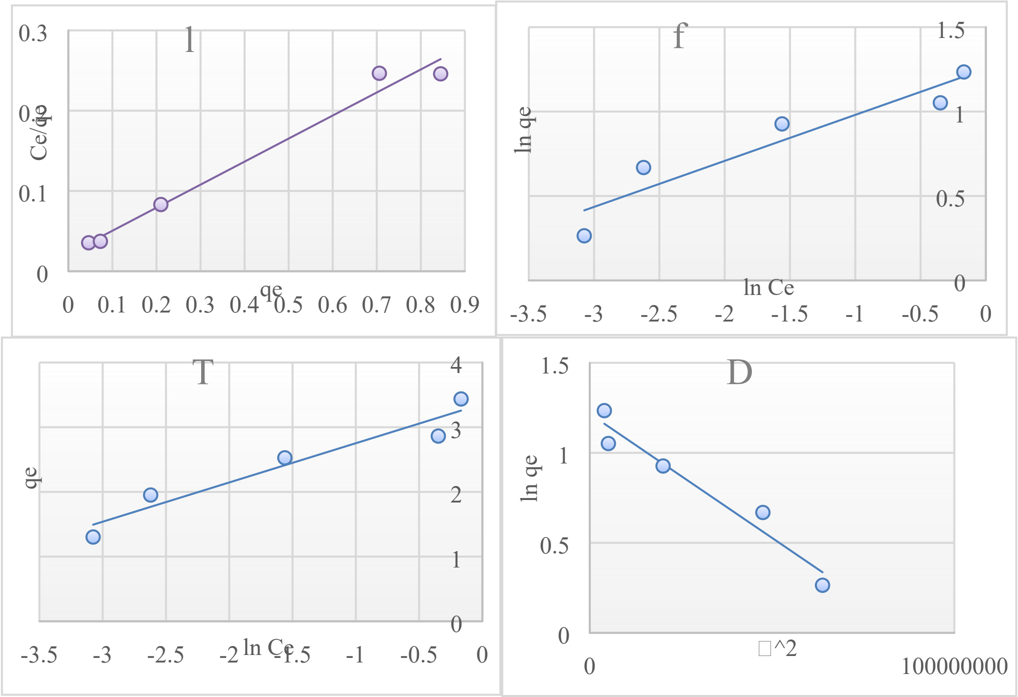

S3 Adsorption isotherm for MG dye

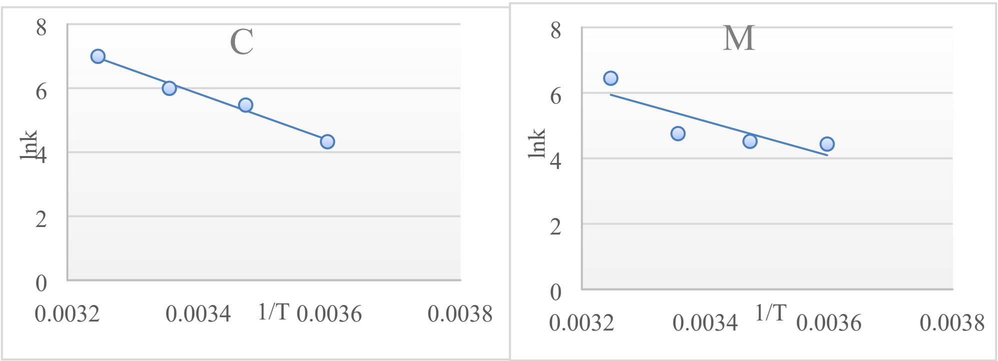

S4 linear relationship of Van't Hoff equation for CV and MG dyes
